# Supplementary material for: Pyruvate dehydrogenase kinase 1 controls triacylglycerol hydrolysis in cardiomyocytes
Source: J Biol Chem. 2025 Mar 10;301(4):108398. doi: 10.1016/j.jbc.2025.108398 (PMC11999607; doi:10.1016/j.jbc.2025.108398)
Supplement: Supplementary figures [file mmc1.pdf]

Pyruvate dehydrogenase kinase 1 controls triacylglycerol hydrolysis in cardiomyocytes

Michael G. Atser, Chelsea D. Wenyonu, Elyn M. Rowe, Connie L. K. Leung, Haoning Howard Cen, Eric D. Queathem, Leo T. Liu, Renata Moravcova, Jason Rogalski, David Perrin, Peter Crawford, Leonard J. Foster, Armando Alcazar, James D. Johnson*

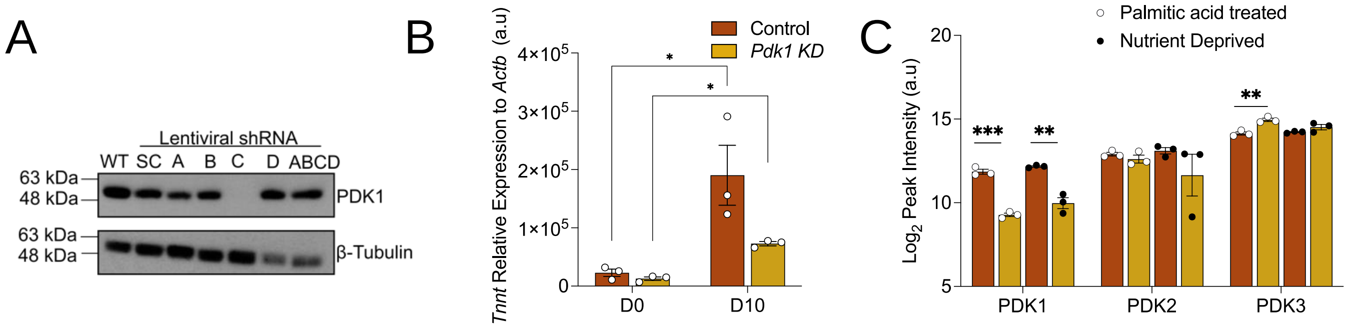

Figure S1: Model Validation. (A) Representative Western blot of PDK1 protein abundance following transduction of H9c2 myoblast with four lentiviral shRNA (A, B, C (*Pdk1* KD), D) targeting *Pdk1* and a scrambled shRNA (SC) as control. Untransduced cells are denoted as wildtype (WT). **(B)** Cardiac troponin T (*Tnnt*) gene expression relative to β-actin (*Actb*) in differentiated H9c2 cardiomyocytes measured by reverse-transcription PCR. **(C)** PDK protein abundance measured by LC-MS/MS based proteomics in control and *Pdk1* knockdown (KD) differentiated H9c2 cardiomyocytes following 375 μM palmitic acid treatment and nutrient deprivation in 5.5 mM glucose, serum-free culture media. Data are shown as mean ± SEM and were analyzed by a two-tailed t-test. * $p < 0.05$; ** $p < 0.01$; *** $p < 0.001$; **** $p < 0.0001$.

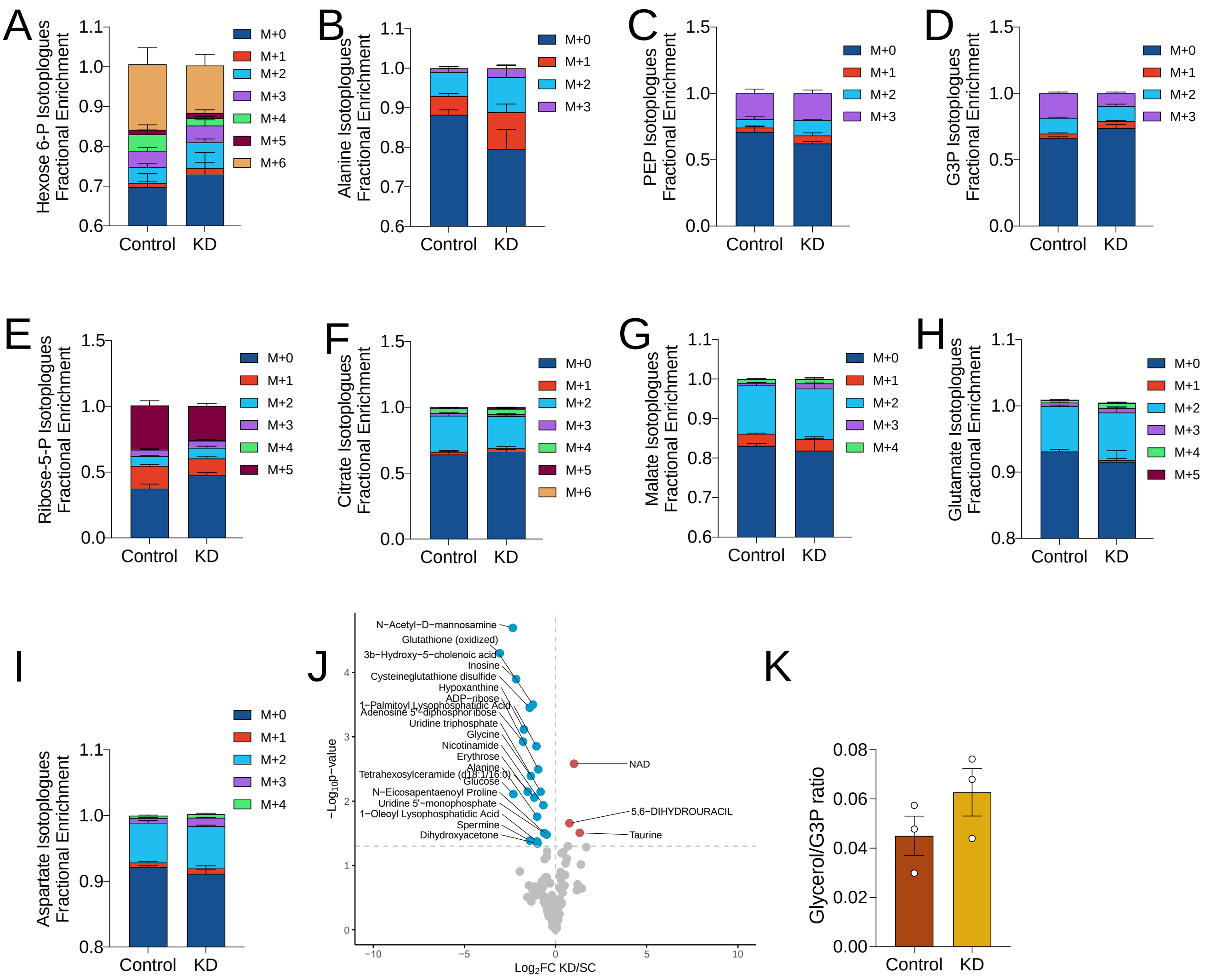

Figure S2: Metabolomics. Cells were treated with palmitic acid and nutrient deprived in low glucose, serum-free media containing 5.5 mM of [U-$^{13}C_6$] glucose for 1 h. Fractional enrichments of **(A)** hexose-6-phosphate, **(B)** alanine, **(C)** phosphoenolpyruvate, **(D)** glyecerol-3-phosphate, **(E)** ribose-5-phosphate, **(F)** citrate, **(G)** malate, **(H)** glutamate, and **(I)** aspartate isotopologues were then assessed. **(J)** Unlabelled glycerol to glycerol-3-phosphate ratio was assessed in unlabelled cells. **(K)** Volcano plot of identified metabolites in cells following 3 h nutrient deprivation. Blue and red dots signify downregulated and upregulated metabolites in *Pdk1* knockdown cells relative to control. Data are shown as mean ± SEM and were analyzed by Limma.

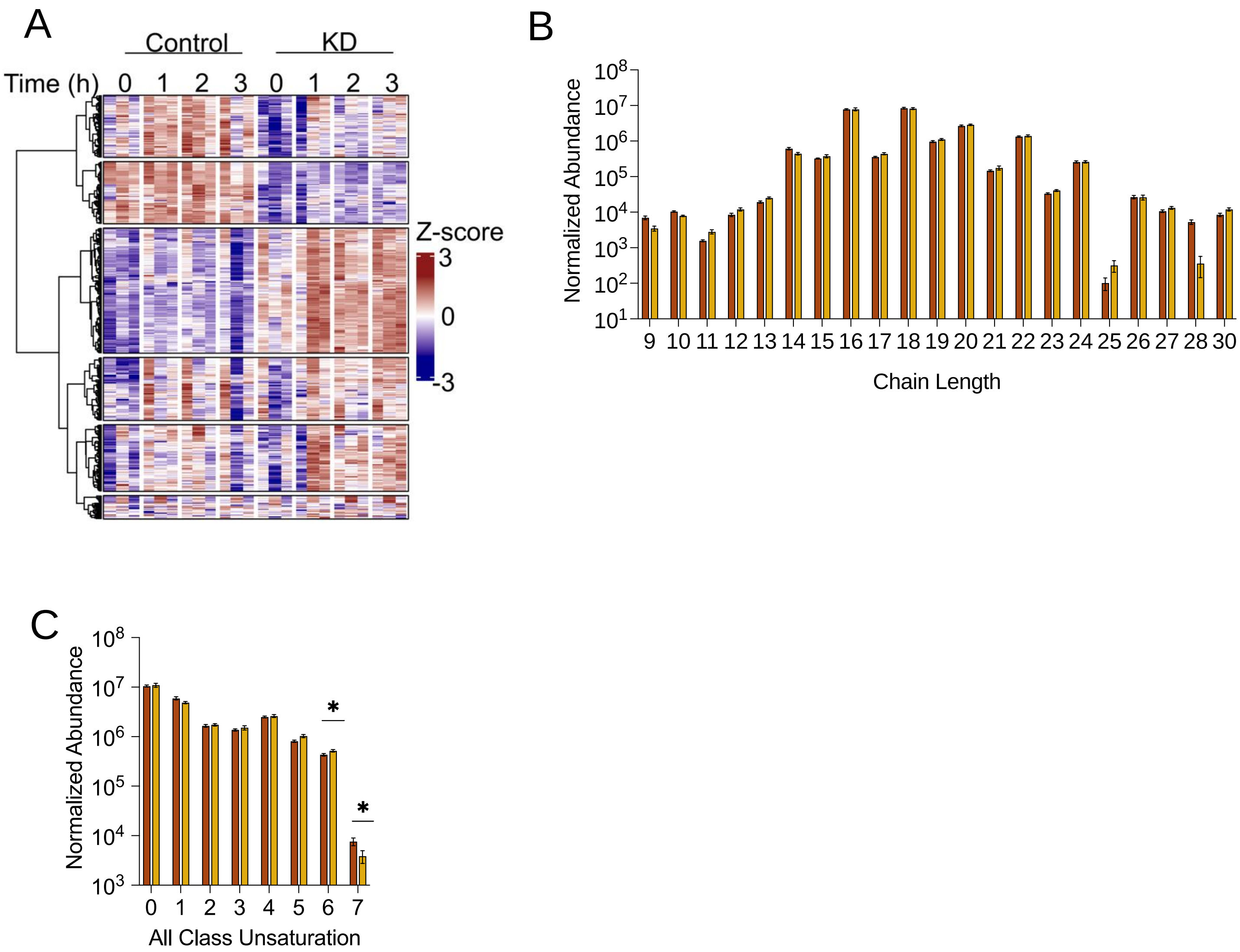

Figure S3: Lipidomics. (A) Heatmap of identified lipids from cells treated with 150 μM palmitic acid and nutrient deprived in low glucose, serum-free media for 3 h. Sum abundance of all lipid species of certain **(B)** chain length and **(C)** unsaturation in cells following palmitic acid treatment**.** Data are shown as mean ± SEM and were analyzed by multiple t-test with Bonferroni-Dunn correction. * $p < 0.05$; ** $p < 0.01$; *** $p < 0.001$; **** $p<0.0001$.

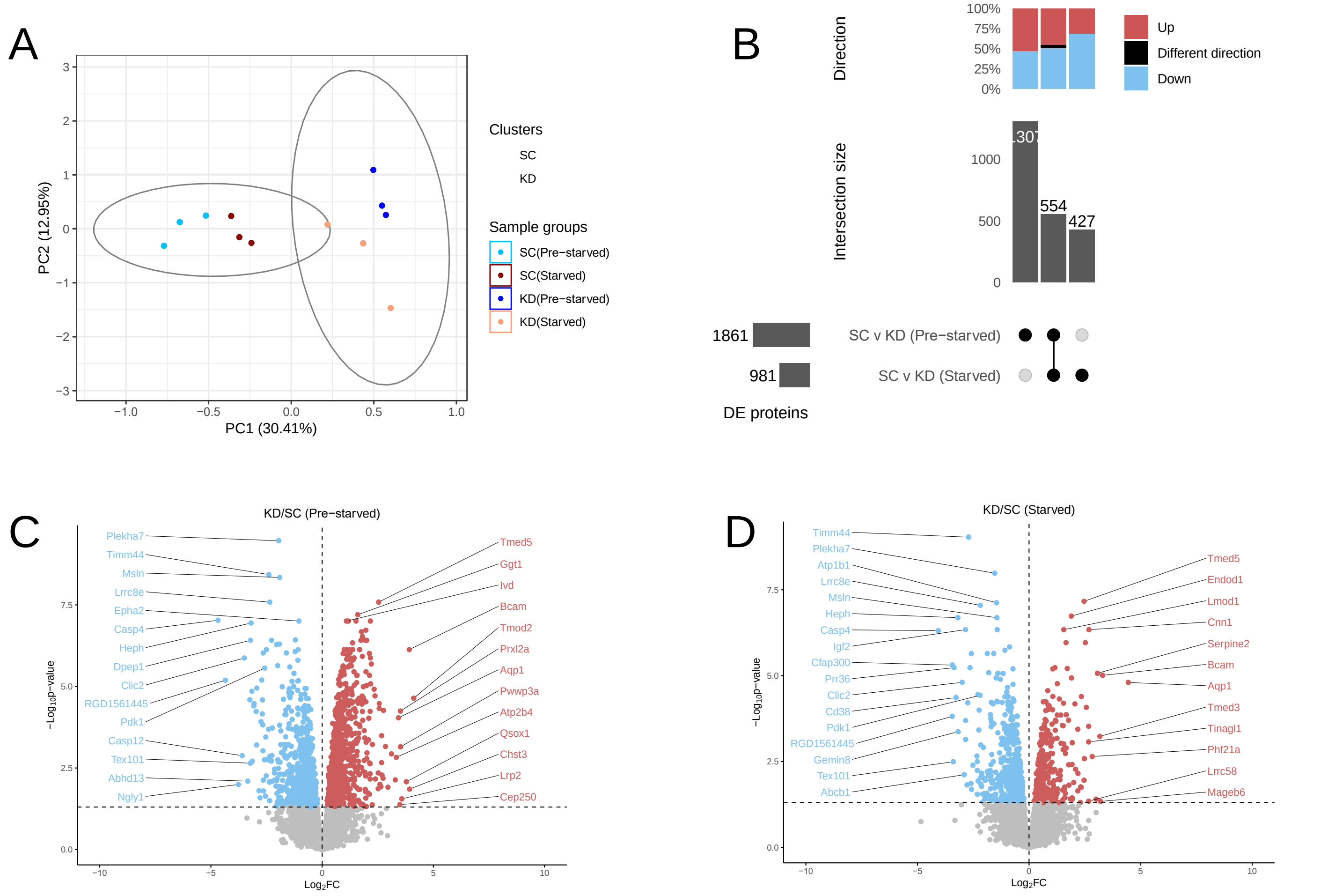

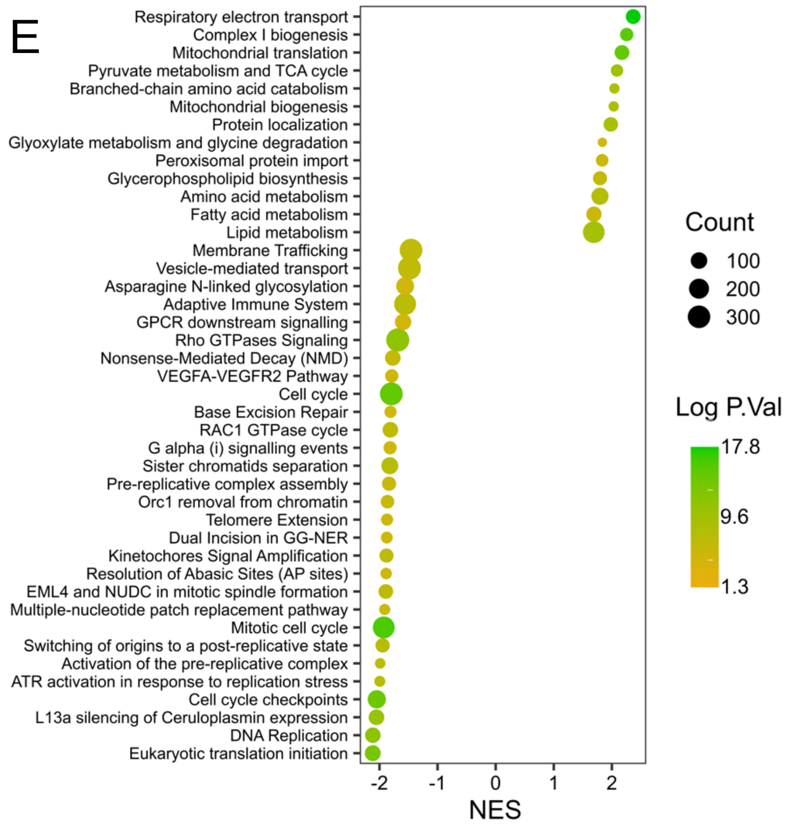

Figure S4: Proteomics. Mass-spec based proteomics was run on cells treated with 375 μM palmitic acid for 18 h (pre-starved), then nutrient deprived in low glucose, serum-free media (starved) for 3 h. Identified proteins were analyzed by **(A)** Principal component analysis, **(B)** UpSet plot to show the intersection of differentially expressed proteins in *Pdk1* knockdown cells relative to control in pre-starved and starved conditions, and volcano plot in **(C)** pre-starved and **(D)** starved conditions. **(E)** Pathway enrichment analysis was then carried out on differentially expressed proteins in *Pdk1* knockdown cells relative to control based on gene-set enrichment analysis derived from the Reactome database.

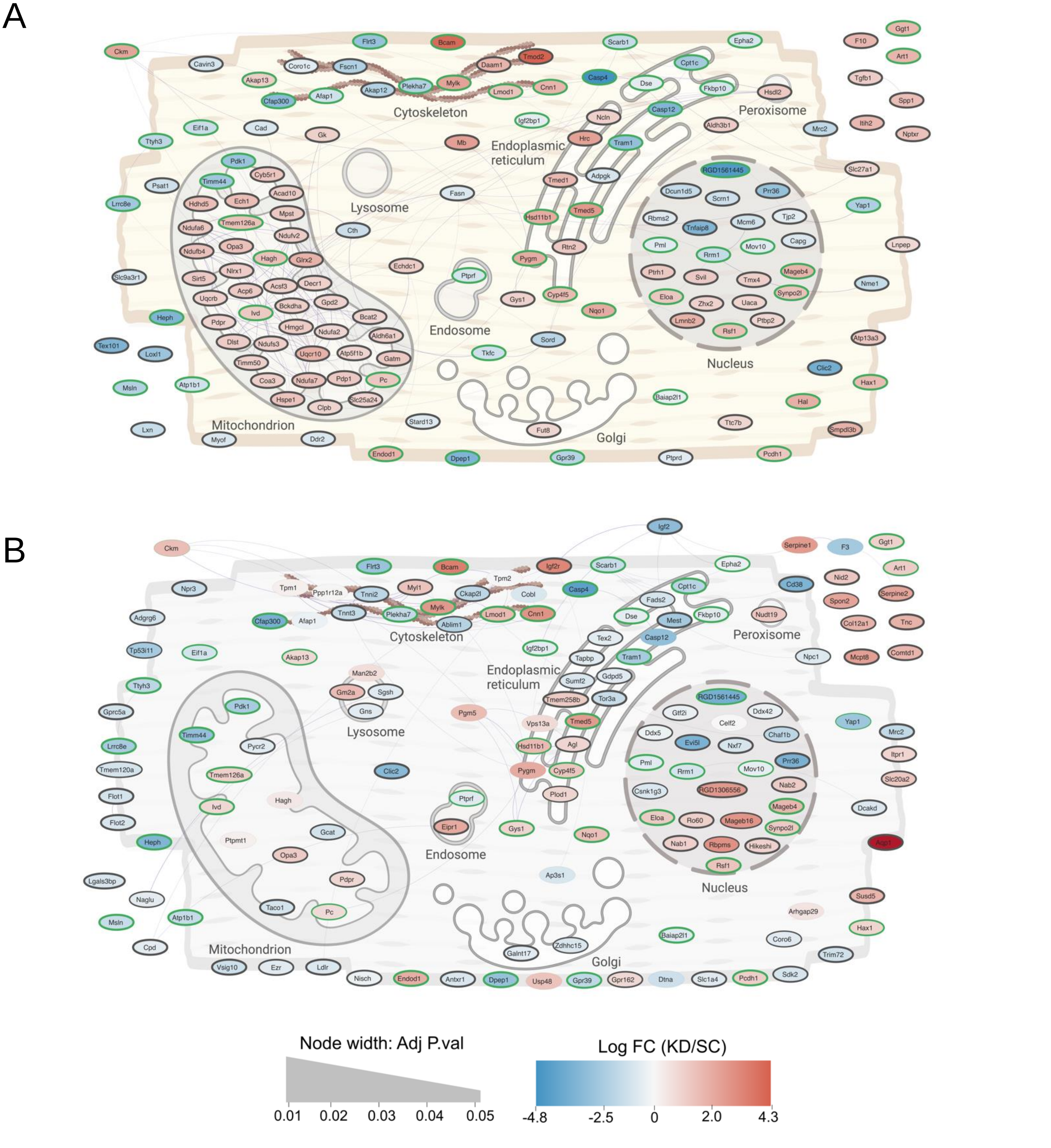

Figure S5: Subcellular mapping of top 150 proteins. Highest confidence (top 150 lowest p-values, per condition) differentially abundant proteins between control and *Pdk1* knockdown cells treated with palmitic acid were connected in a medium-confidence (functional score 0.50) protein-protein interaction network (purple lines) using STRING and depicted in the context of their sub-cellular locations. The color of the nodes represents the fold change in *Pdk1* knockdown cells relative to control, while the thickness of the line around the nodes represents the adjusted p-value, and a green node border illustrates a common protein present in both conditions.

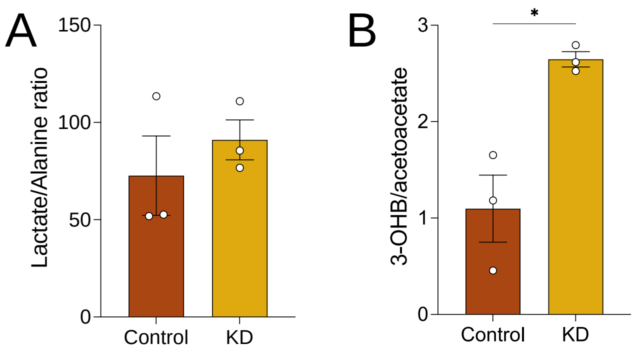

Figure S6: Assessments of Cytosolic and Mitochondrial Redox States. Cells were treated with palmitic acid and nutrient deprived in low glucose for 3 h. Metabolite ratios using low confidence-scored annotated metabolites. A high confidence threshold was set at a Progeneis QI score of ≥45. (A) Lactate (score = 40.3) to alanine (score = 49.3) ratio to assess cytosolic redox state and (B) 3-hydroxybutyrate (score = 40) to acetoacetate (score = 36.3) to assess mitochondrial redox state. Data are shown as mean ± SEM and were analyzed by Two-tailed t-test. * $p < 0.05$; ** $p < 0.01$; *** $p < 0.001$; **** $p < 0.0001$.
